# When Central Tolerance Fails: Thymic Malignancies at the Intersection of Cancer Immunity and Autoimmunity

**DOI:** 10.3390/cancers18050747

**Published:** 2026-02-26

**Authors:** Matthew Abikenari, John Choi, Iman Enayati, Andrew Tucker, Keshav Bhatnagar, Yijiang Chen, Vratko Himic, Justin Liu, George Nageeb, James Poe, Sophia Joy Ong, Vivek Sanker, Markus Diehl, Viktoria Szeifert, Azusa Terasaki, Laura M. Prolo, Edgar Engleman, Derick Okwan-Duodu

**Affiliations:** 1 Department of Neurosurgery, Stanford University School of Medicine, Stanford, CA 94305, USA; 2 Nuffield Department of Clinical Neurosciences, University of Oxford, Oxford OX1 2JD, UK; 3 Department of Orthopaedic Surgery, University of California, Los Angeles, CA 90095, USA; 4 Department of Pathology, Stanford University School of Medicine, 300 Pasteur Dr. Lane Research Building, L200, Stanford, CA 94305, USA; 5 Department of Radiation Oncology, Stanford University School of Medicine, Stanford, CA 94305, USA; 6 Department of Neurosurgery, University of Miami Miller School of Medicine, Miami, FL 33136, USA; 7 Department of Genetics, Stanford University School of Medicine, Stanford, CA 94305, USA

**Keywords:** thymoma, thymic epithelial tumors, central tolerance, autoimmunity, myasthenia gravis, immune checkpoint inhibitors, PD-1/PD-L1, immune-related adverse events

## Abstract

Thymic malignancies are rare cancers arising from thymic epithelial cells and are characterized by a highly diverse clinical phenotype, substantial histologic and morphologic heterogeneity, and frequent associations with autoimmune syndromes. Although the clinical, immunological, and cytoarchitectural changes associated with thymomas have been increasingly elucidated in the contemporary literature, very few studies have interrogated the direct role of tumor staging and histological grading in shaping autoimmunity burden and infection risk. In this narrative review, we synthesize contemporary clinical, immunological, and morphologic evidence linking thymic architecture and selection defects to the spectrum of paraneoplastic autoimmunity (MG, pure red cell aplasia, Good’s syndrome) and to infectious vulnerability. We further appraise emerging therapeutic strategies, including immune checkpoint inhibition and adoptive cellular approaches, through a patient-stratified lens, emphasizing efficacy signals, immune-related adverse events, and practical considerations for selection and monitoring. We conclude by highlighting priorities for future investigation, including refining autoantibody signatures; mapping thymic microenvironments that drive tolerance failure, and prospectively evaluating stratified immunotherapeutic regimens that balance benefit with immune-mediated risk.

## Simple Summary

Thymic epithelial cell tumors are rare malignancies of the thymus, an organ that is crucial in the establishment of central immune tolerance through the deletion of autoreactive T cells. In the context of thymoma, this process of immune tolerance is impaired, resulting in the escape of autoreactive immune cells from the thymus. This has significant consequences in the form of a very high incidence of autoimmune phenomena, such as myasthenia gravis, as well as immunodeficiency disorders like Good’s syndrome, which predispose patients to infections. In this review, we will discuss how architectural and molecular anomalies in the thymus contribute to autoimmunity and immunodeficiency. In addition, we will discuss the implications of this in terms of patient management. We will also discuss new therapeutic approaches, such as immune checkpoint inhibitors, although it must be borne in mind that these agents are associated with a higher risk of severe immune toxicity in this patient population.

## 1. Introduction

The thymus is a lymphoid organ vital for the proper development, differentiation, and proliferation of T lymphocytes that coordinate key adaptive immune responses throughout life. In addition, the thymus establishes immune self-tolerance (central tolerance) through coordinated positive and negative selection, thereby preventing autoimmunity. Thymic malignancies are rare tumors arising from thymic epithelial cells (collectively, thymic epithelial tumors [TETs]) and are associated with diverse clinical presentations, significant histologic and morphologic heterogeneity, and a high prevalence of associated paraneoplastic autoimmune diseases [1,2,3]. This heterogeneity, together with frequent immune sequelae, complicates classification, risk stratification, and management.

Thymoma, the most frequent thymic tumor of the mediastinum, is tightly linked to systemic autoimmunity via disruptions of thymic architecture and T cell selection, affecting multiple systems, organs, and tissues and predisposing to opportunistic infections and additional paraneoplastic autoimmune syndromes [1,3]. Myasthenia gravis (MG) is the most common of such autoimmune manifestations, a long-term neuromuscular junction disease, presenting in roughly 50% of patients with thymoma [4,5]. However, only ~15% of MG patients develop thymoma [6]. Other associated autoimmune syndromes are individually uncommon (roughly 1–5% of patients per illness) and include pure red cell aplasia (PRCA), parathyroid adenoma, hypogammaglobulinemia (Good’s syndrome), autoimmune hepatitis, syndrome of inappropriate antidiuretic hormone secretion (SIADH), and various other paraneoplastic syndromes [7,8]. Thymomas follow an unpredictable natural history and disease progression, ranging from long asymptomatic phases with incidental discovery on chest X-ray/CT performed for unrelated reasons, to aggressively malignant courses [5,9]. Although the exact etiology and oncogenesis of thymomas remain incompletely defined, there has been substantial progress over the last few decades in understanding the cellular, molecular, and dysregulated pathways involved in the pathogenesis of thymoma and its associated paraneoplastic autoimmunity [4,10].

In this narrative review, we synthesize current mechanistic, translational, and clinical evidence on immune dysregulation in TETs, delineate the spectrum of paraneoplastic autoimmunity and infectious vulnerability, and appraise the therapeutic landscape (conventional therapies, immune checkpoint inhibition, and adoptive cellular approaches). We emphasize patient-stratified decision-making and practical implications for diagnosis and multidisciplinary management.

## 2. Methods

This manuscript was conducted as a narrative review with the aim of integrating recent mechanistic and translational insights into central tolerance disruption and immunotherapeutic considerations in thymic epithelial tumors. Given the heterogeneity of this domain and the field’s rapid evolution, we did not conduct a systematic review; instead, we applied a targeted literature search strategy.

We searched PubMed for English-language records from January 2010 to October 2025 using combinations of: “thymic epithelial tumors,” “thymoma,” “thymic carcinoma,” “autoimmunity,” “myasthenia gravis,” “Good’s syndrome,” “hypogammaglobulinemia,” “immune checkpoint inhibitors,” “PD-1,” “PD-L1,” “CTLA-4,” “paraneoplastic,” and “adoptive cell therapy.” Reference lists of key primary studies and recent reviews were screened for additional studies. Preclinical/clinical trials and high-impact conceptual overviews were selectively included to provide a balanced, up-to-date perspective.

We included English-language studies that (a) interrogated autoimmune regulator/Fez family zinc finger 2, negative selection, major histocompatibility complex class II/costimulation, thymic epithelial cell lineages (cortical thymic epithelial cell/medullary thymic epithelial cell), or regulatory T cell biology in thymic epithelial tumors; (b) reported clinical autoimmune or immunodeficiency phenotypes (e.g., myasthenia gravis, pure red cell aplasia, Good’s syndrome) in thymoma/TC; or (c) presented prospective or notable retrospective immunotherapy data in TETs with relevance to immune safety. Studies focused solely on non-thymic autoimmunity without a thymic neoplasm, non-English publications with adequate English syntheses available, or purely in vitro work lacking tolerance relevance were deprioritized. Screening was performed independently by two reviewers with consensus resolution.

From each included study we abstracted: (1) mechanistic variables (AIRE/Fezf2 status, MHC-II/CD80/86, cTEC/mTEC signatures, evidence of negative-selection defects, and Treg differentiation/abundance); (2) phenotype variables (syndrome type, prevalence/when it appears, key diagnostic cues, and a concise management note); (3) immunotherapy variables (setting/phase, ORR, PFS/OS where salient, any-grade and grade ≥3 irAE rates, standout toxicities, e.g., myocarditis/myositis, and baseline autoimmunity allowances); and (4) infection/Good’s variables (risk drivers and practical mitigations such as IVIG, vaccination, and antimicrobial prophylaxis). Evidence was integrated via thematic, narrative synthesis, and organized mechanics-to-management. Given the rarity and heterogeneity of studies, no quantitative meta-analysis was planned.

To enhance usability for clinicians and investigators, we compiled two summary tables directly from extracted items: Table 2 (paraneoplastic spectrum in TETs: syndrome, features, diagnostic cues, management, representative citation) and Table 3 (immunotherapy in TETs: regimen, setting/phase, number of patients, ORR, ≥G3 irAE, notable toxicities, selection/exclusions, note). We also include a definitional table summarizing the WHO histologic classification (Table 1).

Lastly, to operationalize the mechanics-to-management approach, the results and review sections were organized to parallel the extraction domains above. Section 2.1, Section 2.2, Section 2.3 and Section 2.4 provide mechanistic and clinicopathologic evidence supporting the role of thymic epithelial tumor architecture and thymic epithelial cell programs in the development of autoimmune and immunodeficiency syndromes, along with brief epidemiologic and staging information. Section 2.5 integrates the spectrum of paraneoplastic syndromes and infectious susceptibility, including Good’s syndrome and anti-cytokine autoantibodies, into practical screening and prevention issues. Section 2.6 reviews the potential and high-likelihood retrospective immunotherapy evidence in thymic epithelial cell tumors, focusing on response criteria and immune-related adverse events with risk-informed monitoring and selection issues. Finally, Section 2.7 offers a brief conceptual model of the comparison between the immunosuppression in thymoma and the immunosuppression in other cancers, providing context for the potential for checkpoint modulation to be associated with both response and high immune toxicity in the context of immunologic tolerance disorder.

### 2.1. Mechanistic Basis for Tolerance Failure in Thymic Epithelial Tumors

Central tolerance is the primary defense against autoreactivity. Thymocytes first pass positive selection in the cortex. They then enter the medulla, where high-affinity self-recognition results in deletion or diversion to a regulatory lineage. Medullary thymic epithelial cells (mTECs) enforce this process through broad self-antigen display that depends on AIRE and on efficient MHC class II presentation with support from dendritic cells. This architecture enables deletion of harmful clones and the generation of functional regulatory T cells that maintain quiet immune tone after export to the periphery. These steps are well described in contemporary syntheses of thymic biology and tolerance [11,12].

Thymomas imitate thymic tissue yet lack a competent medulla. Most tumors contain large cortical-type epithelial areas rich in immature thymocytes. Medullary maturation is often arrested. This pattern is reproducible across common WHO B subtypes and mixed AB tumors, and it is visible by routine histopathology with corroborating immunophenotypes [13]. The result is a permissive milieu for positive selection with weak or absent negative selection. The tumor thus supports T cell output but fails to censor self-reactive clones. Thymic carcinomas show little thymopoiesis and are rarely associated with paraneoplastic autoimmunity, which fits this model of failed medullary function in thymomas but not carcinomas [14].

AIRE expression falls in many thymomas, and MHC class II is reduced on tumor epithelium. Together, these changes undercut promiscuous gene expression and antigen presentation that are needed for robust deletion in the medulla. Studies that compare subtypes report higher AIRE levels in type A tumors and lower levels in B3 tumors, which aligns with the relative scarcity of autoimmunity in medullary-pattern tumors and the higher frequency in cortex-dominant tumors [15,16]. This biochemistry has clinical echoes. Patients with thymoma often display features that parallel APECED, including anti-cytokine antibodies against type I interferons and Th17 axis cytokines. These antibodies link to infection susceptibility and mucocutaneous candidiasis, and they point back to a shared AIRE-dependent pathobiology [17,18].

Defects in the regulatory arm compound the problem. Thymomas tend to generate fewer FoxP3-positive regulatory T cells than normal thymus or even hyperplastic thymus from non-thymoma MG. The imbalance is seen both in tissue studies and in blood, where Treg frequency and function fall in thymoma cohorts [19,20]. A companion signal is the expansion of Th17 cells with higher IL-17 outputs in MG-associated thymoma. This shift toward Th17 with fewer Tregs favors persistent autoreactivity and supports B cell help [21]. The combined picture is a factory that exports many naïve T cells with insufficient censoring and with weak regulatory restraint.

Antigen supply within the tumor also matters. Thymomas lack normal myoid cells, yet they often express muscle and neuronal antigens or fragments, including acetylcholine receptor subunits, titin, and ryanodine receptor. This atypical repertoire can prime T cells and may foster mimicry that expands clones with cross-reactivity at the neuromuscular junction. These observations help explain why MG dominates the autoimmune spectrum in thymoma and why striational antibodies are common in this subgroup [22].

High-dimensional experimental methods sharpen this framework. Single-cell studies identify mixed cortical and medullary epithelial programs within the same tumor, along with immature medullary subsets that do not achieve full AIRE competence. Spatial transcriptomics shows that most of the mass is cortex-like, yet small islands with medulla signals can support germinal center activity and T follicular helper cells near AIRE low epithelium. These niches could promote local autoantibody maturation and intratumoral antigen presentation in a non-tolerogenic context [23,24]. The architectural and transcriptional maps thus converge with older histology. Positive selection proceeds and negative selection stalls. Treg output falls. Antigen signals persist in the wrong context. The export that follows carries an autoimmune imprint that is strongest in thymomas that resemble cortex and weakest in carcinomas that lack thymopoiesis [13,14].

These mechanisms also inform therapy. Tumors with higher MHC class II and B7 costimulation show inflamed microenvironments and may respond to checkpoint blockade, yet the baseline defect in central tolerance raises the hazard of severe immune toxicity in thymoma. This tension between activity and risk is consistent with prospective series that report responses with prominent neuromuscular and cardiac immune events. Whilst mechanistic biomarkers such as AIRE level, MHC class II density, or Treg scarcity have potential value for selection and monitoring, clinical validation is still in its early days [3,16].

Taken together, the mechanistic story is coherent. Thymomas reproduce positive selection. They truncate negative selection. They release naïve T cells with autoreactive specificities. They reduce the regulatory buffer, and they offer ectopic antigens in a stimulatory setting. These signals lead to myasthenia gravis, pure red cell aplasia, Good’s syndrome, and a set of rarer autoimmune and infectious phenotypes that define the clinical burden of thymic malignancy in practice [3,22].

### 2.2. Epidemiology

Thymoma and related thymic epithelial malignancies are rare, with an overall incidence of approximately 0.13–0.31 per 100,000 person-years and accounting for roughly 0.3–1.5% of all malignancies [8,25]. In Europe, the crude incidence of malignant thymoma is estimated at ~1.4 cases per million per year, corresponding to approximately 1000 new cases annually across the continent based on RARECAREnet data. Comparable incidence rates have been reported in North America using population-based registries such as SEER, which consistently classify thymoma as an ultra-rare cancer [25].

Population studies from East Asia suggest slightly higher reported incidence rates than those observed in Western cohorts, particularly in Japan, Korea, and China, although differences in case ascertainment, histologic classification, and registry practices may contribute to this variation. Across regions, most studies report no meaningful sex predilection, with similar incidence rates in men and women [8,25,26,27,28].

Incidence increases with age and peaks in midlife, typically between 45 and 55 years; a large EUROCARE-4 analysis reported a mean age at diagnosis of 56 years [8,27]. Similar age distributions have been observed in North American and Asian cohorts. Thymoma is uncommon in children and young adults, although cases occur across the age spectrum [8,27]. Notably, incidence rises despite progressive physiological thymic involution with aging, underscoring the paradoxical biology of thymic tumorigenesis in an organ that regresses over time [8,27].

### 2.3. Clinical Presentation, Staging, and Treatment

Clinical presentation is heterogeneous and reflects the combined effects of tumor size, local invasion, and immune dysregulation [5,28,29,30,31]. Many patients are asymptomatic, and the mass is detected incidentally on chest imaging obtained for unrelated conditions [29,30]. A substantial fraction is present through paraneoplastic autoimmunity, most often myasthenia gravis [5,31]. Others come to attention with compressive symptoms from an anterior mediastinal mass, including cough, chest pain, dyspnea, and signs of superior vena cava obstruction [7,30]. These patterns mirror the dual identity of thymoma as a neoplasm and as an instigator of immune dysregulation, which explains why neuromuscular evaluation and thoracic imaging often intersect during the initial workup, and WHO histologic subtype definitions are summarized in Table 1 [31,32].

**Table 1:** WHO classification of Thymoma [33].

**A**	Thymic tumor with an oval shape, absence of both nuclear atypia and non-neoplastic lymphocytes
**AB**	Thymic tumor with foci resembling characteristics of thymoma type A, mixed with the presence of lymphocyte-rich foci
**B1**	Thymic tumor resembling characteristics of a normal and functional thymus, with an expansive appearance of normal thymic cortex and thymic medulla
**B2**	Thymic tumor in which neoplastic epithelial cells are present in the shape of scattered cells with vesicular nuclei and distinct nucleoli, saturated in a large pool of lymphocytes
**B3**	Thymic tumor characterized by the predominance of epithelial cells with round and polygonal shapes and displaying no nuclear atypia
**C**	Thymic tumor is characterized by the absence of immature lymphocytes, a clear sign of cytologic atypia, and the presence of features no longer unique to thymic malignancies, but rather features comparable to carcinomas of other organs

Staging in routine practice relies on the Masaoka–Koga system, which orders disease by capsular invasion and dissemination and remains widely used in clinical series and registries. Stage I lacks invasion, Stage II includes microscopic transcapsular invasion or invasion into mediastinal fat or pleura, Stage III indicates macroscopic invasion of adjacent organs, Stage IVA denotes pleural or pericardial dissemination, and Stage IVB denotes lymphogenous or hematogenous metastasis [29,34]. Survival tracks with stage and with the completeness of resection in historical cohorts, with five-year overall survival near 90 percent in Stage I and near 50 percent in Stage IV, while intermediate stages fall between these anchors [34,35].

Complete surgical resection remains the cornerstone of care for resectable thymoma and is the principal approach to achieve durable control when feasible [34,36]. Thymectomy, defined as complete surgical resection of the thymus gland, is the standard operative strategy in this setting [36]. Despite successful resection, approximately 15–30 percent of patients experience recurrence after a disease-free interval of roughly 60–80 months [36,37]. In patients with advanced Masaoka–Koga stage or unfavorable WHO histology, neoadjuvant therapy is frequently used to improve resectability, including chemotherapy and, in selected cases, radiotherapy based on institutional practice [38,39]. The standard chemotherapeutic regimen for thymoma remains cisplatin, doxorubicin, and cyclophosphamide (CAP) [38,39].

Management of these patients is multidisciplinary and is often shared between oncology and neurology, given the frequent coexistence of autoimmune disease [31,32]. For myasthenia gravis, maintenance immunosuppression with agents such as azathioprine, rituximab, or methotrexate is used to control symptoms and to stabilize the perioperative course when needed [40,41,42]. Close planning around extubation and respiratory monitoring is essential in generalized disease and during periods of fluctuating neuromuscular weakness [40,42].

Contemporary oncologic advances in advanced or refractory thymic malignancies include targeted therapies such as tyrosine kinase inhibitors and mTOR inhibitors, and immunotherapies such as immune checkpoint inhibitors and tumor-associated antigen vaccines [39,43,44,45]. Although recent studies have examined autoimmunity, antibody signatures, and the safety of checkpoint blockade in thymic tumors, few have evaluated in detail how autoimmune presentations, tumor histology, and antibody profiles shape opportunistic infection risk and patient-stratified clinical trajectories [32,45,46].

### 2.4. Pathophysiology, Autoimmunity, and Systemic Vulnerability

Thymic epithelial tumors arise from thymic epithelial cells and display marked histologic heterogeneity that maps to WHO subtypes [3,22]. Type A tumors contain spindle or oval neoplastic cells with few lymphocytes, while type B tumors contain large numbers of immature lymphocytes and recapitulate cortical architecture; type AB tumors combine these features [3,22]. The WHO scheme classifies by microscopic criteria and complements the Masaoka–Koga system, which stages by invasion and spread; Table 1 in this manuscript summarizes the WHO histologic definitions.

Because the thymus is essential for T lymphocyte maturation and central tolerance, thymomas are closely linked to paraneoplastic autoimmunity through disordered selection and atypical thymopoiesis [3]. Thymoma tissue can support intratumoral thymopoiesis that yields CD4 and CD8 lineages, yet selection checkpoints are abnormal and favor export of autoreactive clones [3,47].

In addition, this paradigm of ectopic, dysregulated cellular programming within tumors parallels broader oncologic principles in which aberrant transcriptional and epigenetic regulation, such as lncRNA-mediated state stabilization described in endocrine-resistant breast cancer, drives maladaptive cell fate decisions and therapeutic resistance across disease contexts, which is critical to further examine in the context of thymomas [48].

Type B tumors generate more lymphocytes than type A tumors and show the strongest association with autoimmunity, which aligns with their cortex-like architecture and with defects in antigen presentation that are relevant to selection quality [3,45,49].

Aberrant antigen presentation contributes to this failure of tolerance. Many thymomas display low or absent MHC class II on tumor epithelium, which undermines presentation for both positive and negative selection and weakens the education of CD4 lineage cells [3,49]. This defect can deplete the pool of appropriately selected helper cells and alter crosstalk with B cells in peripheral lymphoid organs, which heightens risk for autoantibody formation and chronic inflammation [3,45].

Regulatory control is also impaired in thymomas. Regulatory T lymphocyte generation is compromised, reducing peripheral restraint of autoreactive T cells and supporting unchecked cytokine signaling and T cell help to B cells [3,50]. This loss of regulation aligns with clinical observations of broad autoimmunity and with immune-mediated toxicities that emerge when checkpoint pathways are therapeutically blocked in thymoma [32,45].

Defective negative selection is also a central feature. Many thymomas show reduced expression of the autoimmune regulator AIRE, which limits promiscuous self-antigen display in medullary compartments and allows self-reactive thymocytes to escape deletion [3,4,51,52]. Negative selection is critical for durable tolerance because it deletes high-affinity self-reactive clones and supports a self-tolerant peripheral repertoire; when this step fails, autoreactive cells seed the periphery and drive disease [14,53]. Clinical series that link low AIRE and selection defects to autoimmune phenotypes in thymoma support this mechanism and explain the frequency of overlapping syndromes in practice [31,45].

Myasthenia gravis illustrates these links. Nearly half of thymoma patients develop myasthenia gravis, which reflects shared antigen targets and convergent antibody signatures across thymoma-associated and non-thymoma myasthenia gravis [3,45,54,55]. Both groups often carry striational antibodies against titin and ryanodine receptor, which track with disease severity and support a model of antigen spread from thymic sources and skeletal muscle targets [56]. Detection of thymoma-associated antibody profiles in early-onset myasthenia gravis can increase suspicion for thymic malignancy and prompt directed imaging in neurology practice [3,55,56,57].

Important caveats remain in the pathophysiology of these tumors. Some type B1 tumors express AIRE yet still associate with robust autoimmunity, which indicates that tolerance failure is multifactorial and may involve the balance of epithelial programs, dendritic cell function, cytokine networks, and the regulatory compartment [51]. Clinical and serologic profiles in thymoma can also differ from those observed in monogenic AIRE deficiency, which suggests overlapping but non-identical pathways to autoimmunity in these settings [32,45]. Despite progress, relatively few studies have tested whether tumor histology, autoantibody panels, and selection biomarkers can predict recurrent infections or composite autoimmune burden across WHO and Masaoka–Koga grades, which leaves a gap for prospective work [32,45].

**Figure 1:**
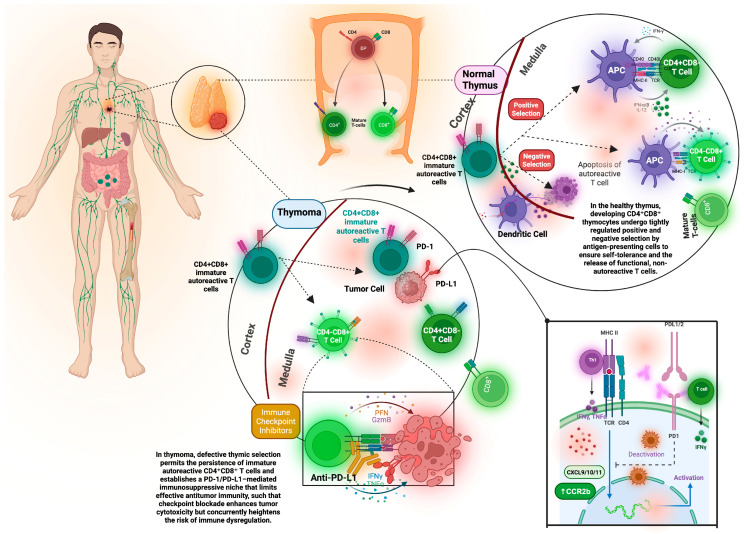
Divergent thymic selection programs in health and thymoma define the intersection of cancer immunity and autoimmunity. In the normal thymus (**Right**), developing CD4+CD8+ double-positive thymocytes undergo precisely regulated processes of both positive and negative selection mediated by thymic epithelial cells and antigen-presenting cells for the induction of deletion of high-affinity autoreactive clones and exportation of functional and self-tolerant CD4+ and CD8+ T-lymphocytes into peripheral tissues. In thymoma (**Middle**), thymic disorganization and impaired negative selection permit the survival and exportation of immature autoreactive CD4+CD8+ T-lymphocytes, thus providing an immunologic milieu of dysfunctional central tolerance and disruption of T-lymphocyte development homeostasis. In the tumor microenvironment, the PD-1/PD-L1 pathway characterizes an immunosuppressive microenvironment, facilitating reduced antitumor cytotoxic immunity and dysregulating T-lymphocyte development and function. Immunologic checkpoint therapy (**Bottom Inset**) may reset dysregulated antitumor cytotoxic immunity; however, it may also enhance autoreactive immune responses, leading to clinical consequences of evolving grade immune-related adverse events. This figure emphasizes thymoma’s unique role in both antitumor immunity responses and the development of autoimmune disease, underscoring its clinical importance for patient-specific immunologic therapies. Figure made with Biorender (December 2025).

The immunologic cascade that follows selection failure reinforces itself through persistent autoreactive T cell export, dendritic cell activation, cytokine skewing, and germinal center activity that amplifies pathogenic antibodies (Figure 1) [54]. Figure 1 recapitulates the divergent thymic selection programs that define the intersection of cancer immunity and autoimmunity.

### 2.5. Paraneoplastic Spectrum and Infectious Vulnerability in Thymic Epithelial Tumors

Paraneoplastic autoimmunity is a defining feature of thymoma; it shapes both diagnosis and long-term outcomes in clinical practice [3,22]. The spectrum is broad, yet it is anchored by myasthenia gravis, which affects a large minority of patients with thymoma and rarely occurs with thymic carcinoma [3,14]. This distribution mirrors the biology of cortex-dominant thymomas that export insufficiently censored T cells and support autoantibody formation against neuromuscular junction targets [3,13].

Hematologic autoimmunity is less common but clinically consequential. Pure red cell aplasia presents with severe anemia and reticulocytopenia and often responds to calcineurin inhibition after tumor management, which suggests a T cell-mediated pathogenesis that persists outside the primary tumor bed [39,58]. Reported series place prevalence in the low single digits among unselected thymoma cohorts, which underlines the need for focused screening only when cytopenias or symptoms arise [39,58].

Good’s syndrome presents with thymoma alongside adult-onset hypogammaglobulinemia and a near absence of circulating B cells. It carries substantial risk for recurrent sinopulmonary and opportunistic infections together with autoimmunity [59,60]. Cellular studies show reduced naïve and memory CD4 subsets and impaired helper function, which explains both infection susceptibility and the paradox of coexisting autoimmune disease in some patients [60,61]. The clinical backbone of care is regular intravenous immunoglobulin replacement with careful vaccination planning that avoids live vaccines once significant immunodeficiency is documented [59,61].

Neurologic paraneoplastic disease extends beyond myasthenia gravis. Thymoma associates with peripheral nerve hyperexcitability syndromes, including acquired neuromyotonia and Morvan syndrome, which often involve antibodies to CASPR2 or LGI1 and present with cramps, fasciculations, dysautonomia, and occasionally encephalopathy [62,63]. Early recognition permits effective immunotherapy and tumor control, which together reduce relapse risk and long-term morbidity [62,63].

Autoimmunity also involves skin, liver, and endocrine organs in smaller proportions of patients, which reflects broad defects in tolerance rather than a single tissue target [32,64]. These syndromes demand organ-specific management plans that coordinate with oncologic treatment and with neurology if myasthenia gravis coexists [31,32].

Patients with thymoma are also subject to increased infectious vulnerability, which arises from two convergent pathways. First, there is combined immunodeficiency as seen in Good’s syndrome with hypogammaglobulinemia, lymphopenia, and poor vaccine responses, which lead to recurrent and opportunistic infections such as candidiasis and cytomegalovirus [59,61]. Secondly, there are anti-cytokine autoantibodies that mirror monogenic AIRE deficiency and neutralize type I interferons, as well as Th17 cytokines, which predispose to severe viral and fungal diseases, including chronic mucocutaneous candidiasis [17,18]. These mechanisms can coexist, and they explain both the breadth and severity of infections in selected patients with thymoma [17,60].

The management of these paraneoplastic phenomena follows these mechanisms. Patients with hypogammaglobulinemia should receive periodic intravenous immunoglobulin and prompt antimicrobial therapy for suspected bacterial disease alongside individualized consideration of prophylaxis in those with recurrent infections [59,61]. Vaccination programs should prioritize inactivated vaccines and avoid live agents when cellular or humoral defects are present, and should be timed away from planned cytotoxic or B-cell depleting therapy to improve responses [59,61]. Clinicians should consider screening for anti-interferon and anti-Th17 pathway antibodies in patients with unusual viral illness or chronic candidiasis because these markers point to specific risks and may guide the need for aggressive infection control strategies [17,18]. Coordination with neurology, hematology, and infectious disease is essential when overlapping syndromes exist [31,32].

The synthesis of these data supports a simple principle. Thymoma generates autoreactivity through disordered selection and reduced regulation, and it disables key elements of host defense in a subset of patients, which together create a wide and sometimes hazardous clinical spectrum that demands structured screening and early supportive therapy (Table 2) [3,22].

### 2.6. Immunotherapy in Thymic Epithelial Tumors: Efficacy and Safety

Immune checkpoint blockade has meaningful activity in a subset of thymic epithelial tumors, yet the risk of severe immune toxicity is higher than in many other solid tumors and is most pronounced in thymoma [3,32,51]. This pattern reflects high baseline antigenicity and defective central tolerance in thymoma together with abundant costimulatory and antigen presentation programs in selected tumors [14,16].

Pembrolizumab has reproducible efficacy in pretreated thymic carcinoma with objective response rates approaching 20% with durable benefit in a fraction of patients [65,66]. In the landmark single-arm phase II study in refractory thymic carcinoma, the objective response rate was 22.5% with manageable toxicity in most patients and with a signal that higher PD-L1 expression correlated with response [65]. A multicenter Korean phase II study that enrolled both thymic carcinoma and a small number of thymomas reported an objective response rate of nearly nineteen percent in carcinoma and confirmed activity with careful patient selection [66]. These results established anti-PD-1 therapy as an option in advanced carcinoma after chemotherapy [65,66].

Nivolumab has more limited single-agent activity in thymic carcinoma. In the Japanese PRIMER phase II study, the objective response rate was zero with frequent disease stabilization and a disease control rate that approached seventy percent [67]. This divergence from pembrolizumab may reflect cohort differences or biomarker distributions, and it underscores the need for biomarker-guided selection in small populations [16,67].

Anti-PD-L1 therapy has shown striking activity in thymoma with prominent immune toxicity. In the NIH phase I trial, avelumab achieved responses in the majority of the thymoma cohort, and severe immune-related events reported included myositis, myocarditis, and myasthenia gravis-like syndromes [14]. These observations align with the thymoma-specific risk implied by central tolerance failure [3,32].

Combination strategies that pair checkpoint blockade with antiangiogenic therapy have improved response metrics at the cost of added toxicity. The CAVEATT single-arm phase II trial of avelumab plus axitinib in type B3 thymoma and thymic carcinoma reported a 34% objective response rate with grade 3 or higher adverse events in 34% of patients; hypertension and transaminase elevations were common, and immune-mediated myositis was uncommon but clinically important [68]. The PECATI open-label phase II trial of pembrolizumab plus lenvatinib in platinum-refractory type B3 thymoma and thymic carcinoma reported a 36% objective response rate with grade 3 or higher treatment-related adverse events near 29%, most often hypertension and mucosal toxicities with immune effects consistent with PD-1 blockade [69]. These studies provide contemporary benchmarks for activity with careful eligibility that generally excludes uncontrolled autoimmune disease and often requires close cardiac and neuromuscular monitoring [68,69].

Prospective series and focused correlative studies point to plausible biomarkers for selection and monitoring. PD-L1 expression enriches for response in some cohorts, although thresholds and assays vary across trials [65,66]. Expression of CD80 and CD86 and MHC class II on tumor epithelium correlates with immune activation signatures and may predict benefit from immune checkpoint blockade in unresectable or recurrent disease [16]. These signals fit the broader model of antigen presentation and costimulation driving both efficacy and toxicity in thymoma and selected carcinomas [14,51].

Safety remains the limiting factor in thymoma. Rates of severe immune-related adverse events such as myositis, myocarditis, and myasthenia gravis exacerbation exceed those seen in most tumor types and often occur early in treatment [14,32]. Baseline autoantibodies against acetylcholine receptor and striational antigens together with low B cell counts mark a higher risk in thymoma and should prompt multidisciplinary evaluation and close surveillance if immunotherapy is pursued [32]. These principles motivate cautious selection, strict baseline assessment, and rapid treatment interruption at first signs of neuromuscular or cardiac involvement in routine practice [32,45].

Immune checkpoint inhibitors offer clinically meaningful options in advanced thymic carcinoma and in selected thymomas, but success depends on disciplined selection, biomarker-aware monitoring, and early management of immune toxicity in populations with inherent tolerance defects [14,16,32,65]. The trial landscape that supports these conclusions is summarized in Table 3.

Care is safest when evaluation maps the autoimmune and immunodeficiency phenotypes at baseline and when treatment is sequenced with clear guardrails for perioperative and immunotherapy risks [3,31]. Three domains anchor this approach: phenotype screening, perioperative myasthenia management, and infection prevention in patients with hypogammaglobulinemia or anti-cytokine autoantibodies.

Baseline screening should document autoimmune burden and immune competence before definitive treatment. History and examination should probe fatigable weakness, bulbar symptoms, dysautonomia, rashes, cytopenias, and recurrent infections, since these findings will direct further testing and facilitate early subspecialty input [31,62]. Laboratory assessment should include a complete blood count, quantitative immunoglobulins, and lymphocyte subsets with attention to B cell depletion that flags Good’s syndrome [59,61]. Serology should include acetylcholine receptor binding and modulating antibodies with striational antibodies to titin and ryanodine receptor in suspected thymoma-associated myasthenia gravis, because these markers correlate with severity and guide perioperative planning [56,57]. When the history suggests unusual viral disease or chronic mucocutaneous candidiasis, testing for anti-interferon and anti-Th17 axis autoantibodies can clarify risk and focus infection mitigation [17,18]. Cardiac and neuromuscular baselines are useful if immunotherapy is planned and should include creatine kinase, high-sensitivity troponin, a resting electrocardiogram, and echocardiography in patients with symptoms or risk factors, with neurology and cardiology consultation when prior autoimmune disease is present [32].

Perioperative management for thymectomy is safest when myasthenia gravis is under control. Patients with generalized disease benefit from optimization with anticholinesterase therapy and, when needed, a short course of corticosteroids or steroid-sparing agents to stabilize strength before surgery [40,41]. Intravenous immunoglobulin or plasma exchange is appropriate in selected patients with bulbar or respiratory involvement or with prior crises to reduce postoperative respiratory complications [5,40]. Anesthesia and critical care teams should anticipate variable neuromuscular transmission and plan for cautious extubation with close monitoring in the first twenty-four to forty-eight hours after surgery [40]. Long-term immunosuppression with azathioprine, methotrexate, or rituximab remains standard for refractory or relapsing myasthenia and should be coordinated with oncologic therapy [41,42].

Prevention of infection is central in thymoma patients with hypogammaglobulinemia or combined defects. Good’s syndrome requires periodic intravenous immunoglobulin to reduce serious bacterial infections and to improve quality of life, with dose and interval individualized to maintain protective trough IgG levels [59,61]. Vaccination should prioritize inactivated formulations and avoid live agents once significant cellular or humoral defects are confirmed; timing should be optimized before cytotoxic therapy or B cell depletion to improve responses [59,61]. Antimicrobial prophylaxis is individualized and is most often considered for patients with recurrent sinopulmonary infections or for those receiving prolonged corticosteroids or lymphocyte-depleting agents [59,61]. When anti-interferon or anti-Th17 pathway autoantibodies are present, clinicians should maintain a low threshold for early antiviral therapy or antifungal prophylaxis during periods of immunosuppression [17,18].

Selection and monitoring for immune checkpoint therapy require added caution in thymoma. Baseline autoantibodies against acetylcholine receptor or striational antigens and low peripheral B cell counts identify a subgroup at higher risk for severe neuromuscular toxicity with PD-1 or PD-L1 blockade [32]. In thymic carcinoma, activity outweighs risk in many patients, but careful screening remains prudent because overlap syndromes can occur [65,66]. Early detection protocols during the first two cycles should include interval symptom checks for dyspnea, diplopia, dysphagia, and myalgias, with repeat creatine kinase and high-sensitivity troponin at least every one to two weeks for the first six to eight weeks, then as clinically indicated [32]. Suspected myocarditis or myositis warrants immediate interruption of immunotherapy and initiation of high-dose corticosteroids, with escalation to intravenous immunoglobulin or plasma exchange if myasthenic features or steroid-refractory weakness emerge [32]. Multidisciplinary management with neurology and cardio-oncology improves outcomes and limits irreversible toxicity [32].

This management architecture links directly to the biology outlined earlier. Disordered selection and reduced regulatory tone create a baseline of high autoimmune risk, while syndromic immunodeficiency in a subset raises infection hazards. Upfront phenotyping, vigilant perioperative care, and prophylaxis where indicated can reduce complications and improve tolerance of oncologic therapy without compromising tumor control [3,31].

### 2.7. Conceptual Analog of Opposing Immunological Axes: An Example

A direct conceptual analog to the immune logic of autoimmunity in the context of thymomas, impaired tolerance programming due to dysfunctional antigen presentation and lack of regulatory oversight, also applies to the immune microenvironment in glioblastoma (GBM), but in the form of extreme immunosuppression rather than autoimmunity [3,70,71,72]. 

Whereas thymomas export inadequately censored, autoreactive T cells due to failed central tolerance [3], GBM constructs a peripheral tolerance niche within the tumor microenvironment, dominated by suppressive myeloid populations and dysfunctional antigen presentation [73,74,75]. Tumor-infiltrating macrophages and neutrophils in GBM present antigens in the absence of costimulation but in the presence of metabolic or cytokine-mediated constraints to force infiltrating T cells to undergo exhaustion, anergy, or deletion [76,77,78]. In the case of both conditions, immune impairment is due not to the absence but to dislocated mechanisms of immune tolerance, contributing to the lackluster response to immune checkpoint blockade therapy in situations where the immune foundation is inherently perturbed [1,2,3,79,80,81].

The tolerance-promoting program in GBM is further backed by the glioma-neuronal pair, which adds a novel immune conditioning that exists solely within the central nervous system and differs from immune environments in metastases. Neuronal function and neurotransmission control the state of myeloid cells and solidify T cell metabolic exhaustion, as if the thymoma’s expression of ectopic antigens existed in a non-pathologic situation [82,83,84]. The common theme is the role of a neuroimmune tolerance circuit, wherein exposure, regulation by the CNS, and metabolic suppression interact synergistically to inhibit productive immunity. Breaking this circuit by immune checkpoint-targeted therapy or other immune-modulatory regimens poses the risk of revealing underlying inflammatory disease, as occurs in the thymoma model [14,16,85,86,87]. The view of GBM as a model of pathologic immune education and immune ignorance has a role for a common interpretive framework regarding resistance and neurotoxicities for the neuro-oncologist and the role of central tolerance for the immunologist [16,88,89].

It should be noted that this comparison is meant as a conceptual framework rather than a disease-specific parallel, as a way to discuss how seemingly opposite failures of immune education, as seen with central tolerance failure in thymoma and enforced peripheral tolerance in GBM, can create resistance and unusual patterns of toxicity with immunotherapy. The context of thymoma as part of this tolerance-axis model will be used to discuss how immune checkpoint blockade in tolerance disorders creates therapeutic responses and unusual patterns of immune toxicity.

Collectively, these similarities cast thymoma and glioblastoma as the dual sides of the same coin: mechanistically linked models of disordered immune education, one defined by failed central tolerance and the other by enforced peripheral tolerance. Collectively, all these provide insights into the immunologic link connecting neuro-oncology.

## 3. Limitations of the Evidence Base

The literature on thymic epithelial tumors remains constrained by rarity and by heterogeneous study designs, which complicates inference on epidemiology, autoimmunity, and infection risk [8]. Most reports are single-center and retrospective with small sample sizes, inconsistent follow-up, and variable definitions of autoimmune and infectious outcomes, which limits generalizability and the precision of risk estimates [31,62]. Many cohorts lack standardized capture of ethnicity and geographic origin despite signals that incidence and presentation vary across Asian and Pacific Islander populations and across European regions, which restricts external validity and comparative analyses [8]. Clinical heterogeneity before definitive surgery introduces additional confounding since patients may receive immunosuppressants, chemotherapy, or radiotherapy that alter immune phenotypes, antibody profiles, and infection susceptibility at baseline or in the perioperative period [31,32]. Furthermore, the emergent use of artificial intelligence and large language models in clinical oncology has opened a new venture in which pathology images can be trained to predict immunotherapy responses across numerous cancers and autoimmune syndromes [90,91], leading the direction of precision medicine in immunology and clinical oncology.

Lastly, temporal relationships between myasthenia gravis and thymoma are also difficult to resolve because autoimmune manifestations may precede tumor detection by years, which obscures causal attribution for downstream infections and for changes in antibody repertoires after thymectomy [47,62]. Prospective data that link WHO histology and Masaoka–Koga stage to the subsequent burden of autoimmune comorbidity and to recurrent infections are scarce, and very few studies integrate selection biomarkers such as AIRE status or MHC class II with clinical outcomes, which leaves a gap between mechanism and prognosis [13,16,32].

### 3.1. Subtype-Resolved Tolerance Failure and Risk-Stratified Immune Management in Thymic Epithelial Tumors

#### 3.1.1. WHO Subtype–Linked Disruption of Cortical vs. Medullary Epithelial Programs

With respect to the immunopathology of the various WHO subtypes of thymomas, the best way to conceptualize the autoimmune syndrome is by considering the balance between the programs that drive the processes of thymopoiesis/positive selection in the cortex and the programs that facilitate the processes of negative selection and the production of Treg cells in the medulla. Immunophenotyping of the subtypes of thymomas based on the expression of cortical TEC markers such as β5t/PRSS16/cathepsin V and medullary epithelial markers such as AIRE/CD40/claudin-4 indicates that type B thymomas are more cortical-leaning, while the expression of the latter is decreased in the progression towards the more aggressive subtypes. In particular, the expression of AIRE was reported to be higher in type A than in type B3 thymomas, supporting the idea that type B3 is less capable of censoring autoimmunity [92].

#### 3.1.2. irAE Risk Stratification for Checkpoint Blockade by Histology and Baseline Immune State

Clinical patterns of toxicity are emerging to suggest that thymoma is no longer a homogeneous risk category for ICI therapy, where higher-grade thymomas (typically B2/B3) are enriched for immune toxicity in myocarditis-centric studies, again reflecting a baseline defect in tolerance that is unmasked by immune checkpoint inhibition. Thymoma-specific correlative studies have now shown that baseline positivity for anti-acetylcholine receptor antibodies and baseline B-cell lymphopenia are correlated with subsequent avelumab-induced myositis, often in association with neuromuscular cardiac irAEs, and a practical approach to stratifying thymoma patients by histology and baseline autoantibody status is therefore proposed [93,94].

#### 3.1.3. A Practical Prophylaxis Algorithm for Good’s Syndrome (Vaccines, Antimicrobials, IVIG)

Infection risk in Good’s syndrome is well-characterized, and a simple approach to standardize current clinical practice is as follows: (1) confirm diagnosis by measuring levels of immunoglobulins and quantifying lymphocyte subsets; (2) use IVIG to replace missing antibodies and adjust dose and frequency according to clinical infections and IVIG trough levels; (3) use inactivated vaccines and avoid live vaccines in patients with clinically significant immunodeficiency; (4) use antimicrobials to prevent infections in patients with recurrent sinopulmonary infections, opportunistic infections, and additional immunosuppression; and (5) monitor infections and immune status over time, escalating prophylactic measures during periods of increased risk such as during chemotherapy or prolonged use of corticosteroids [95].

Recent high-resolution chromatin profiling efforts have further clarified the systems-level mechanisms by which thymic epithelial cells (TECs), particularly medullary TECs, mediate central tolerance. A recent 2025 Nature article established that TECs specifically utilize epigenetic noise or stochastic variability in chromatin accessibility to express ectopic tissue-restricted gene products necessary for negative selection broadly [96]. Perhaps most importantly, this process was shown to be AIRE-independent with respect to chromatin accessibility but instead correlated with the regulated inactivation of the tumor suppressor gene p53, nucleosome instability, and AT-rich sequence content conducive to transcriptional variability. In this manner, tolerance is not mediated by deterministic gene expression programs but rather by controlled levels of chromatin instability to increase transcriptional diversity in self-antigen loci, thus expanding the scope of central tolerance education.

This concept is particularly pertinent to thymic epithelial tumors, in which tumor cell development likely disrupts this finely balanced regulation of chromatin stability, TEC cell state plasticity, and tolerance programming. Altered TEC lineage development, epigenetic variability, or regulation of chromatin accessibility in thymomas may result in an insufficiently diverse (and hence conducive to the escape of autoreactive clones) or aberrantly organized self-antigen expression repertoire, which may explain the high incidence of associated autoimmune syndromes. This is particularly relevant in light of recent studies indicating that immune dysregulation in thymomas is not simply mediated by AIRE and MHC-II expression but rather may reflect more fundamental defects in TEC cell state regulation in the tumor microenvironment [92,93,94,95].

## 4. Conclusions and Future Directions

Future work should first resolve disease anticipation and prediction in routine practice to facilitate more targeted patient-specific therapies. Multicenter prospective cohorts should test whether WHO subtype and Masaoka–Koga stage, combined with antibody panels that include acetylcholine receptor and striational antibodies, predict the number and timing of autoimmune comorbidities and the risk of severe or recurrent infections after thymectomy [31,47]. These cohorts should include a standardized collection of ethnicity and geography to quantify incidence gradients seen across Asian and Pacific Islander groups and across European regions and to examine whether immune phenotypes vary by ancestry or environment [8]. Data dictionaries should harmonize definitions for autoimmune events and infections and should predefine time windows relative to surgery and systemic therapy to clarify temporal order and causality [32,62].

Known mechanisms should then be integrated into prognosis. Pathology reports can incorporate AIRE status, MHC class II, and costimulatory molecules such as CD80 and CD86, together with concise notes on cortex and medulla programs, to bridge selection biology with clinical risk models [13,16].

Spatial and single-cell studies already show that cortex-heavy architecture in myasthenia gravis is associated with thymoma with reduced medullary niches. These signatures should be tested prospectively as predictors of autoimmunity and infection burden in parallel with clinical staging [23,24].

Moving forward, therapy should advance with safety at the forefront. Immune checkpoint inhibition provides benefit in pretreated thymic carcinoma and in selected thymomas, but early severe neuromuscular and cardiac toxicity remains a major hazard in thymoma [14,32,65]. Trials should mandate baseline neurology and cardio-oncology assessments, require serology for acetylcholine receptor and striational antibodies, and record B cell counts before the first dose. Early safety blood tests should include serial creatine kinase and high-sensitivity troponin during the first six to eight weeks of therapy. In addition, protocols should embed rapid interruption and high-dose corticosteroids at the first signs of myositis, myocarditis, or myasthenic worsening with escalation to intravenous immunoglobulin or plasma exchange when indicated [32]. Combination regimens such as avelumab plus axitinib or pembrolizumab plus lenvatinib warrant study against single-agent immunotherapy in biomarker-selected populations, with adaptive designs that stop early for futility or excess toxicity [52].

Next, infection prevention should be built into care pathways. Prospective registries should track intravenous immunoglobulin dosing, vaccine responses to inactivated platforms, and the effect of antimicrobial prophylaxis in Good’s syndrome and in patients with anti-interferon or anti-Th17 axis autoantibodies. Outcomes should include hospitalization rates, quality of life, and interactions with oncologic therapy, including checkpoint blockade and B cell depleting agents [17,18,59,61].

Finally, genomic and transcriptomic profiling should be tied to immune phenotypes. Thymic carcinoma shows a higher tumor mutation burden than thymoma and distinct drivers, while thymoma carries recurrent GTF2I mutations with very low mutation burden. Cohorts with paired exome and RNA sequencing and immunophenotyping can test whether these classes align with antigen presentation programs, costimulation, and response or toxicity with checkpoint therapy [14].

## Figures and Tables

**Table 2:** Paraneoplastic spectrum in thymic epithelial tumors (TETs).

Syndrome	Key Features	Diagnostic Cues	Note
Myasthenia gravis (MG) [3]	Fluctuating fatigable weakness; bulbar/ocular involvement common; occurs in ~30–50% of thymomas (rare in thymic carcinoma)	AChR or MuSK antibodies; decrement on RNS; thymoma on imaging	Standard MG therapy (pyridostigmine, corticosteroids, steroid-sparing agents, IVIG/PLEX for crises); oncologic management of thymoma
Good’s syndrome (thymoma with hypogammaglobulinemia) [59]	Adult-onset combined immunodeficiency: low/absent B cells, hypogammaglobulinemia, CD4 lymphopenia; recurrent sinopulmonary/opportunistic infections; coexisting autoimmunity	Marked hypogammaglobulinemia (IgG/IgA/IgM), very low/absent circulating B cells; thymoma present	Lifelong IVIG replacement; aggressive infection prevention (vaccines as appropriate, prompt antimicrobials)
Pure red cell aplasia (PRCA) [58]	Severe normocytic anemia with reticulocytopenia; marrow shows absent erythroid precursors; often co-occurs with thymoma	Profound reticulocytopenia; marrow erythroid aplasia; exclude viral/drug causes	High response to calcineurin inhibitors (e.g., cyclosporine); thymectomy when feasible; monitor for infectious complications
Neuromyotonia/Morvan/limbic encephalitis (LE) [62]	Peripheral nerve hyperexcitability (myokymia, cramps), dysautonomia, insomnia; LE with memory loss/seizures; frequently CASPR2-IgG (±LGI1) and thymoma	Serum/CSF antibodies to CASPR2/LGI1; EMG neuromyotonia; MRI/EEG changes in LE	Tumor control (thymectomy when feasible) plus immunotherapy (steroids, IVIG/PLEX; consider rituximab for relapsing disease)
Hypogammaglobulinemia without full Good’s phenotype [59]	Recurrent infections may precede or follow thymoma diagnosis	Reduced immunoglobulins; variable B cell counts	Consider IVIG if infections/failure to mount vaccine responses; evaluate for concurrent autoimmune cytopenias
Autoimmune hepatitis/endocrinopathies (e.g., thyroiditis, hypoparathyroidism) [32]	Hepatitis (elevated transaminases), endocrine dysfunction; shares mechanistic parallels with central tolerance defects	Autoantibodies (e.g., anti-TPO), abnormal LFTs, and endocrine panels	Organ-specific immunosuppression; coordinate with tumor therapy
Dermatologic autoimmunity (e.g., pemphigus, lichen planus) [64]	Mucocutaneous blisters/erosions; lichenoid eruptions; may accompany or herald thymoma	Tissue/direct immunofluorescence	Dermatology co-management; topical/systemic immunosuppression; address underlying tumor

**Table 3:** Clinical Efficacy and Immune Toxicity of Immune Checkpoint–Based Therapies in Thymic Epithelial Tumors.

Agent	Setting	*n*	ORR	≥G3 irAE	Notable Toxicities	Exclusions	Note
Pembrolizumab (anti–PD-1) [65]	Refractory/metastatic thymic carcinoma; single-arm phase II (single center)	40	22.5%	15%	Severe autoimmune AEs (e.g., myocarditis, hepatitis); pneumonitis	Active autoimmune disease generally excluded; prior lines allowed	Durable responses in a subset; PD-L1 high tumors enriched for response
Pembrolizumab (anti–PD-1) [66]	Refractory TETs (TC n = 26; thymoma n = 7); open-label phase II (multicenter, Korea)	33	~19% (TC)	~15% (all severe AEs)	Autoimmune AEs are higher than in many other tumors	Autoimmune disease excluded	Confirms activity in TC; safety signal warrants caution
Nivolumab (anti–PD-1)—PRIMER [67]	Unresectable/recurrent thymic carcinoma; single-arm phase II (Japan)	24–25	0%	~8–12%	Immune-related AEs (thyroiditis, rash)	Autoimmune disease excluded	Stable disease common (DCR ~70%); limited ORR
Avelumab (anti–PD-L1) [14]	Relapsed thymoma (phase I expansion; NIH)	7	~57%	High	Myositis (often with myocarditis/neuromuscular overlap), thyroiditis; association with baseline AChR-binding Abs and B-cell lymphopenia	Autoimmune disease largely excluded; intensive safety monitoring	Strong activity signal in thymoma offset by high, mechanism-linked toxicity
Avelumab + Axitinib (CAVEATT) [68]	Pre-treated type B3 thymoma and thymic carcinoma; single-arm phase II (Europe)	32	34%	34%	Hypertension, transaminase increased, fatigue; immune-mediated myositis, rare but observed	Standard ICI combination exclusions (active autoimmune disease, uncontrolled CV risk)	VEGFR blockade + PD-L1 shows synergistic activity; manageable but non-trivial toxicity
Pembrolizumab + Lenvatinib (PECATI) [69]	Platinum-refractory B3 thymoma and thymic carcinoma; open-label phase II (Spain)	81	36%	~29%	Hypertension, hypothyroidism, stomatitis; immune AEs per PD-1 class	Active autoimmune disease requiring systemic therapy was excluded	Contemporary benchmark for PD-1 + TKI in TETs; clinically meaningful activity

## Data Availability

No new data were created or analyzed in this study. Data sharing is not applicable to this article.
